# Environmental Persistence and Disinfection of Lassa Virus

**DOI:** 10.3201/eid2911.230678

**Published:** 2023-11

**Authors:** Marlee Shaffer, Robert J. Fischer, Shane Gallogly, Olivia Ginn, Vincent Munster, Kyle Bibby

**Affiliations:** University of Notre Dame, Notre Dame, Indiana, USA (M. Shaffer, O. Ginn, K. Bibby);; National Institutes of Health, Hamilton, Montana, USA (R.J. Fischer, S. Gallogly, V. Munster)

**Keywords:** Lassa virus, viruses, zoonoses, persistence, disinfection, viability

## Abstract

Lassa fever, caused by Lassa virus (LASV), is endemic to West Africa, where ≈300,000 illnesses and ≈5,000 deaths occur annually. LASV is primarily spread by infected multimammate rats via urine and fomites, highlighting the need to understand the environmental fate of LASV. We evaluated persistence of LASV Josiah and Sauerwald strains on surfaces, in aqueous solutions, and with sodium hypochlorite disinfection. Tested strains were more stable in deionized water (first-order rate constant [k] for Josiah, 0.23 days; for Sauerwald, k = 0.34 days) than primary influent wastewater (Josiah, k = 1.3 days; Sauerwald, k = 1.9 days). Both strains had similar decay rates on high-density polyethylene (Josiah, k = 4.3 days; Sauerwald, k = 2.3 days) and stainless steel (Josiah, k = 5.3 days; Sauerwald, k = 2.7 days). Sodium hypochlorite was highly effective at inactivating both strains. Our findings can inform future risk assessment and management efforts for Lassa fever.

Lassa fever is an acute viral hemorrhagic illness caused by Lassa virus (LASV), an enveloped RNA virus of the *Arenaviridae* family (*1*). Lassa fever is endemic to West Africa, causing ≈300,000 illnesses and ≈5,000 deaths annually (*1*,*2*). Approximately 80% of Lassa fever cases result in mild symptoms; however, the remaining 20% are serious infections that cause hemorrhaging and vomiting and simultaneously effect several organs, including the kidneys, liver, and spleen, which can have lifelong effects for survivors (*3*). The overall mortality rate for Lassa fever is 1%; however, patients hospitalized with a severe infection have an increased mortality rate of 15%–20% (*1*,*4*).

The natural reservoir of LASV is the multimammate rat, *Mastomys natalensis*, which is found in 50%–98% of households in West Africa and puts ≈58 million persons at risk for infection (*2*,*3*,*5*). LASV outbreaks have primarily been limited to 2 distinct regions, Nigeria and the Mano River Basin, which comprises areas of Liberia, Guinea, and Sierra Leone (*3*,*6*). The outbreaks in Nigeria were caused by lineages II and III, and the outbreaks in the Mano River Basin were cause by lineage IV (*3*,*6*). Ecologic niche models predicting expansion of suitable environmental conditions for LASV, coupled with population increases in those areas, estimate a 761% increase in the risk for infection among populations in endemic areas (*6*). During 2012–2022, Lassa fever cases have increased, likely because of an increasing population, urbanization, and environmental changes (*7*). Concurrently, the range and number of known lineages have also increased; new lineages were discovered in Mali in 2009 and Benin in 2014 (*8*,*9*). 

Transmission of LASV is primarily through direct contact with fomites contaminated with rat urine or feces or through inhalation (*2*,*10*). Secondary person-to-person transmission can occur from direct contact with bodily fluids of infected persons, specifically in healthcare settings that have inadequate infection prevention and control measures (*11*). Thus, understanding the environmental persistence of LASV is critical for mitigation and control efforts.

The World Health Organization lists LASV as a priority pathogen with pandemic potential because the virus has a relatively long incubation period and populations outside of endemic areas lack prior immunity (*12*). Infected persons shed the virus in bodily fluids for up to 3 months (*10*). No standard practice exists for disposing of LASV infectious waste, leading to concerns about further environmental transmission. 

Limited research evaluating the environmental persistence and disinfection of LASV is available, creating a critical gap in understanding the potential for continued environmental transmission. We investigated the persistence of 2 LASV strains representing lineages II and IV, on surfaces, and in water and wastewater. We then assessed LASV disinfection with sodium hypochlorite to assess a potential LASV management approach.

## Materials and Methods

We used the Josiah strain to represent lineage IV and the Sauerwald strain to represent lineage II. All experiments were conducted in a Biosecurity Level 4 facility. 

### Surface Persistence Experiments

We conducted solid surface persistence experiments similar to those previously described (*13*). In brief, for each time point, we placed three 4-cm diameter disks of either stainless steel or high-density polyethylene (HDPE) into individual wells of a 6-well plate. We evenly distributed 10^6^ 50% tissue culture infectious dose (TCID_50_) of either Josiah or Sauerwald LASV isolates in the cell-free medium on the disks, and allowed the plates to air dry at 20°C. At each time point, we placed 450 µL of Dulbecco’s Modified Eagle Medium (DMEM; Sigma-Aldrich, https://www.sigmaaldrich.com), supplemented with heat-inactivated fetal bovine serum (FBS; GIBCO/ThermoFisher, https://www.thermofisher.com) and 2% Pen/Strep (GIBCO) to a final concentration of 50 U/mL penicillin and 50 µg/mL streptomycin, onto the surface and gently agitated to free the surface-bound virus. Then we added L-glutamine (GIBCO) to a final concentration of 2 mmol in an appropriately labeled 2 mL screw top vial and froze at −80°C. We then determined viral titers for each surface.

### Aqueous Persistence Experiments

We conducted all experiments in triplicate and at 20°C. We added Josiah and Sauerwald LASV stocks to each replicate of deionized water and primary influent wastewater to achieve a final concentration of 10^6^ TCID_50_/mL (Appendix). We collected samples daily for 5 days. At each time point, including zero, we added 50 µL of the aqueous matrix from each of the bulk wastewater or deionized water vials into 450 µL of DMEM (Sigma-Aldrich), modified as described above. Then we added L-glutamine (GIBCO) to a final concentration of 2 mmol in an appropriately labeled 2 mL screw top vial and froze at −80°C. We used 50 µL of the nonspiked aqueous matrix for negative controls. We then determined viral titers for each replicate.

### Aqueous Disinfection Experiments

We used primary influent wastewater for the aqueous disinfection experiments to replicate the type of wastewater in low- and middle-income countries in which emergency disinfection for LASV would be used. We assessed the initial chlorine demand of the wastewater matrix by using the Chlorine (Free and Total) Test Kit (Hach, https://www.hach.com) (*14*). We assessed LASV disinfection in triplicate in wastewater for Josiah and Sauerwald isolates at 1 mg/L, 5 mg/L, and 10 mg/L of sodium hypochlorite. In the top row of a deep-well 96-well plate, we spiked virus into wastewater or deionized water to an initial concentration of 1:1,000,000. We removed an initial sample, diluted 1:10 in DMEM, and froze at −80°C. We then added sodium hypochlorite to each well to achieve the desired sodium hypochlorite concentrations. We took the time zero sampling ≈20 seconds after adding chlorine to enable sample mixing. We removed 50-µL samples at 1, 5, 15, and 30 minutes, mixed with 50 µL of sodium thiosulfate (134 nmol), and concentration matched (w:w) with sodium hypochlorite (40 nmol) to quench any remaining free chlorine. We then added that mixture to 400 µL of DMEM to achieve a final 1:10 dilution before freezing at −80°C until titrated.

### Virus Titration

To assess the concentration of viable virus, we performed TCID_50_ by using 4 10× dilution series for each sample. We then incubated Vero E6 cells with the virus dilutions for 1 h at 37°C, then removed the virus from the 2 highest concentrations, rinsed 2 times with PBS, and added 200 µL of fresh culture medium. We also added 100 µL of fresh culture medium to each of the remaining wells in the plate. We incubated the plates at 37°C for 7 days, inspected for cytopathic effect, and scored plates by using the Spearman-Kärber method.

### Statistical Analysis

We analyzed the decay of infectious LASV strains for all experiments by using a first-order decay model and excluded data points below the limit of detection for the assay, as previously described (*13*–*16*). In the decay models, each plotted point represents the mean of 3 independent experimental replicates run concurrently, and the error bars show the standard deviation. We performed all plotting, regressions, and statistical analyses by using R Studio 2022.07.2 (R Foundation for Statistical Computing, https://www.r-project.org).

## Results

### Persistence of Lassa Virus on Surfaces

**Figure 1:**
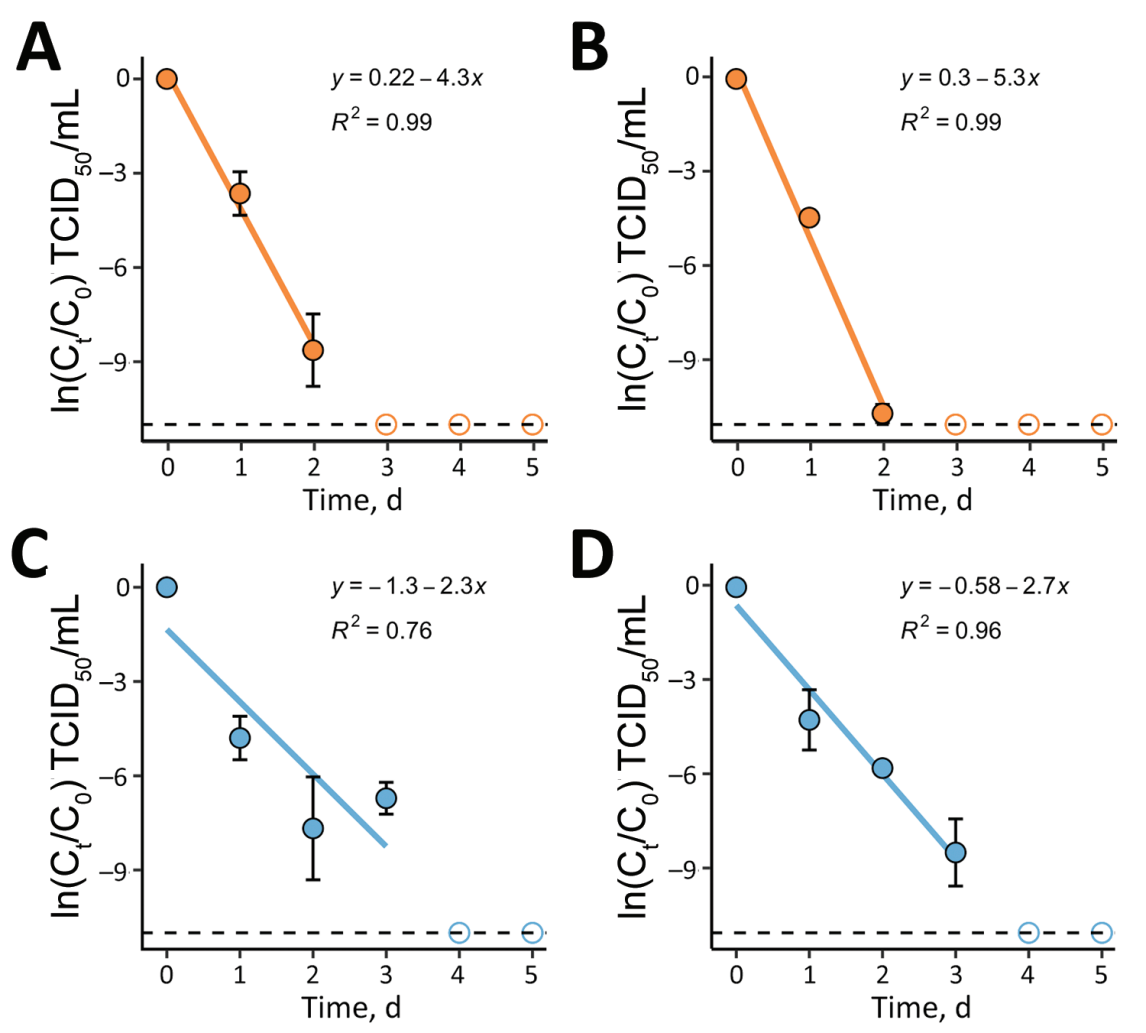
First-order decay of Josiah and Sauerwald isolates on surfaces in a study of environmental persistence and disinfection of Lassa virus. A) Josiah strain on high-density polyethylene; B) Josiah strain on stainless steel; C) Sauerwald strain on high-density polyethylene; D) Sauerwald strain on stainless steel. Each plotted point represents the mean of 3 independent experimental replicates run concurrently, and the error bars show the standard deviation. Solid blue and orange lines indicate decay rates; dashed lines indicate the assay’s detection limits; hollow circles represent points below the detection limit that were not included in the regression analysis. The equation of the linear regression (*y*) and the *R*^2^ value are shown on the plots. All plotting, regressions, and statistical analyses were performed using R Studio 2022.07.2 (The R Foundation for Statistical Computing, https://www.r-project.org). C, initial concentration of infectious virus; C_t_, concentration of infectious virus at time, t; TCID_50_, 50% tissue culture infectious dose.

Stainless steel and high-density polyethylene (HDPE) are commonly used nonporous materials for hospital equipment. In addition, HDPE is a component of common full-body personal protective coveralls and gowns worn in hospitals (*13*). We deposited LASV on HDPE and stainless steel surfaces, allowed concentration to air dry at 20°C, and measured virus viability over 5 days. We found no statistically significant difference in decay between the HDPE and stainless steel within a single strain (Table 1; Figure 1). Conversely, we noted a small but significant difference between the 2 strains on stainless steel (p = 0.028) but not on HDPE.

**Table 1:** First-order decay rate constants and decimal reductions for 2 virus strains on different surfaces in study of environmental persistence and disinfection of Lassa virus*

Measurements	High-density polyethylene			Stainless steel	
	Josiah strain	Sauerwald strain		Josiah strain	Sauerwald strain
*k*, d	4.3 (3.5–5.1)	2.3 (0.50–4.1)		5.3 (4.3–6.3)	2.7 (2.0–3.4)
D value, d	0.54 (0.45–0.66)	1.0 (0.56–4.6)		0.43 (0.37–0.54)	0.85 (0.68–1.2)

*Values are no. (95% CI). D value, time required to kill 90% of virus; *k*, first-order rate constant.

### Persistence of Aqueous LASV

**Figure 2:**
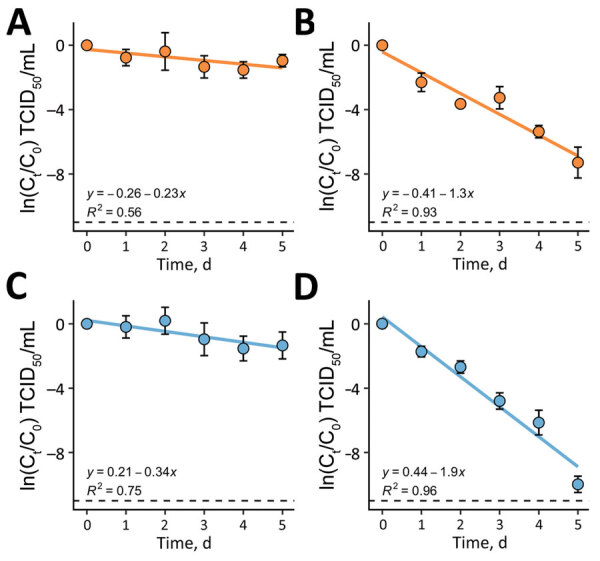
First-order decay of Josiah and Sauerwald strains in water in a study of environmental persistence and disinfection of Lassa virus. A) Josiah strain in deionized water; B) Josiah strain in wastewater; C) Sauerwald strain in deionized water; D) Sauerwald strain in wastewater. Each plotted point represents the mean of 3 independent experimental replicates run concurrently, and the error bars show the standard deviation. Solid blue and orange lines indicate decay rates; dashed lines indicate the assay’s detection limits; hollow circles represent points below the detection limit that were not included in the regression analysis. The equation of the linear regression (*y*) and the *R*^2^ value are shown on the plots. All plotting, regressions, and statistical analyses were performed using R Studio 2022.07.2 (The R Foundation for Statistical Computing, https://www.r-project.org). C, initial concentration of infectious virus; C_t_, concentration of infectious virus at time, t; TCID_50_, 50% tissue culture infectious dose.

We spiked LASV strains into deionized water and raw municipal wastewater and monitored persistence for 5 days. The decay rate was significantly higher in wastewater than deionized water (p<0.002), which can be attributed to multiple factors (*17*). First, microorganisms found in wastewater contribute to viral inactivation (*18*). Second, RNA genomes experience increased degradation when exposed to higher concentrations of ammonia found in wastewater (*19*). We noted no statistically significant difference between LASV strains for deionized water or wastewater (Table 2; Figure 2). 

**Table 2:** First-order decay rate constants and decimal reductions for 2 virus strains in different aqueous solutions in study of environmental persistence and disinfection of Lassa virus*

Measurements	Deionized water			Wastewater	
	Josiah strain	Sauerwald strain		Josiah strain	Sauerwald strain
*k*, d	0.23 (0.04–0.43)	0.34 (0.15–0.53)		1.3 (0.94–1.6)	1.9 (1.5–2.3)
D value, d	10.0 (5.4–57.6)	6.8 (4.3–15.4)		1.8 (1.4–2.4)	1.2 (1.0–1.5)

*Values are no. (95% CI). D value, time required to kill 90% of virus; *k*, first-order rate constant.

### LASV Disinfection with Sodium Hypochlorite

**Figure 3:**
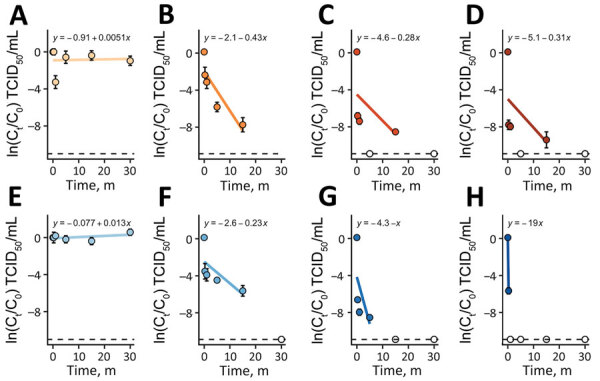
Virus disinfection in a study of environmental persistence and disinfection of Lassa virus. Graphs depict disinfection of Lassa virus with sodium hypochlorite in municipal wastewater. A–D) Josiah strain; E–H) Sauderwald strain. Graphs depict sodium hypochlorite concentrations of 0 mg/L (A, E), 1 mg/L (B, F), 5 mg/L (C, G), and 10 mg/L (D, H). Each plotted point represents the mean of 3 independent experimental replicates run concurrently, and the error bars show the standard deviation. Solid blue and orange lines indicate decay rates; dashed lines indicate the assay’s detection limits; hollow circles represent points below the detection limit that were not included in the regression analysis. The equation of the linear regression (*y*) and the *R*^2^ value are shown on the plots. All plotting, regressions, and statistical analyses were performed using R Studio 2022.07.2 (The R Foundation for Statistical Computing, https://www.r-project.org). C, initial concentration of infectious virus; C_t_, concentration of infectious virus at time, t; TCID_50_, 50% tissue culture infectious dose.

Sodium hypochlorite is a widely available disinfectant is used for wastewater disinfection (*20*). Sodium hypochlorite is effective at inactivating microorganisms and produces a disinfectant residual, which would be advantageous for short-term use in a public health emergency (*20*). Hospitals have also used sodium hypochlorite on site to manage wastewater containing high-consequence pathogens (*21*). Prior LASV disinfection studies have evaluated thermal inactivation, ultraviolet irradiation, and gamma irradiation in human serum and plasma (*22*). We assessed the disinfection kinetics Josiah and Sauerwald LASV strains in raw municipal wastewater by using sodium hypochlorite at 0 mg/L, 1 mg/L, 5 mg/L, and 10 mg/L (Figure 3).

Neither strain was removed at 0 mg/L sodium hypochlorite over the experimental timeframe. Increased sodium hypochlorite concentrations resulted in significantly higher decay rates for Josiah (p = 0.001–0.3) and Sauerwald (p = 0.001–0.007) strains of LASV. The Sauerwald strain showed faster inactivation than the Josiah strain for both 5 mg/L and 10 mg/L (p = 0.04). Those results indicate that sodium hypochlorite effectively inactivates the LASV strains analyzed in this study.

## Discussion

In general, we found LASV persisted longer in aqueous solution than on surfaces. Josiah strain persistence was significantly longer in wastewater than on HDPE and stainless steel (p<0.01). Both Josiah and Sauerwald strains had significantly lower decay rates in deionized water than on HDPE and stainless steel (p<0.01–0.02), indicating higher stability in deionized water than on either surface (Appendix Tables 2–9).

Contact with potentially contaminated surfaces without proper personal protective equipment, especially in hospitals, is anticipated to be a potential transmission route for LASV (*23*). However, previous studies have not analyzed the persistence of infectious LASV on surfaces to support that claim. Quantifying LASV persistence is critical to implementing proper control mechanisms and prevention measures (*24*). The persistence of enveloped viruses on surfaces is specific to the virus, the surface, and the temperature and humidity. A previous study using Ebola virus at 21°C and 40% relative humidity on HDPE and stainless steel found the decay of Ebola was slower than we found for LASV on the same surfaces (*15*). In contrast, another study found the decay rates of SARS-CoV-1 and SARS-CoV-2 on stainless steel were faster than the Sauerwald strain but slower than the Josiah strain of LASV from this study (*25*). The slow decay rates and increased LASV persistence in aqueous solution compared with surfaces seen in our data suggests that efforts are best directed at managing fluids containing infectious LASV recently deposited onto surfaces.

Wastewater in LASV endemic locations will likely be more concentrated than the primary influent wastewater we used in our study. Wastewater from a LASV treatment unit will have higher ammonia levels, total suspended solids, and biologic oxygen demand. Those physiochemical changes would be expected to decrease LASV persistence but increase the amount of sodium hypochlorite needed to reach similar free chlorine levels due to increased chlorine demand. If sodium hypochlorite is used for disinfection, then chlorine residual should be assessed to ensure proper sodium hypochlorite concentrations for LASV inactivation.

Previous research analyzed the persistence of other priority pathogens, including Ebola virus, SARS-CoV-2, and Nipah virus, on representative surfaces or solutions (*14*,*26*,*27**)*. Ebola virus and SARS-CoV-2 persisted in wastewater similarly to LASV under the same experimental conditions (*14*,*26*). A study analyzing Nipah virus in blood and tissue cultures found that decay rates increased in samples that were exposed to the atmosphere, further describing how experimental conditions are critical when discussing the persistence of a virus (*27*). Compared with disinfection of SARS-CoV-2 and Ebola virus, LASV was inactivated faster under the same dose of sodium hypochlorite and all 3 viruses have been shown to be highly susceptible to disinfection with sodium hypochlorite (*15*,*16*).

Bacteriophage Phi6 and murine hepatitis virus are commonly used as surrogates for enveloped viruses of concern, such as Ebola and SARS-CoV-2, and potentially LASV. Bacteriophage Phi6 and murine hepatitis virus decay rates were previously assessed in wastewater (*28*). Compared with the LASV decay rates from this study, surrogate decay was 2–5 times faster than LASV (*28*), indicating surrogate viruses are not representative of LASV persistence in wastewater. Furthermore, in deionized water, the decay rate for bacteriophage Phi6 was faster than we found for LASV, showing lower surrogate virus persistence (*17*). Conversely, in autoclaved wastewater influent, the decay rate of bacteriophage Phi6 was lower than what we found for LASV strains (*17*), suggesting potential differences in the decay of viruses based on the specific matrix. Overall, commonly used surrogates for enveloped viruses of concern do not indicate the behavior of LASV in aqueous solutions.

Given the differences in stability between the surrogates and LASV, the differences in stability and susceptibility to chlorine disinfection between the LASV strains were not predicted here. The 2 strains evaluated displayed strain-dependent behavior, suggesting that the potential for environmental transmission might vary by strain. Those differences could have ramifications that affect virus transmission between the zoonotic reservoir and humans, as well as influencing human-to-human transmission. Future transmission studies should incorporate potential differences in stability between lineages into the study design. Additional investigations into the mechanistic basis of those differences could help elucidate potential regional differences of spillover risk in human populations.

In conclusion, LASV is a World Health Organization priority pathogen with pandemic potential. Our findings supply data for the environmental persistence of LASV on representative surfaces, in aqueous solutions, and with sodium hypochlorite disinfection. LASV showed shorter persistence on surfaces than previously assessed enveloped virus surrogates and priority pathogens, and longer persistence in aqueous solution. LASV was rapidly disinfected in the presence of free chlorine from the addition of sodium hypochlorite. The data we provide on the stability and control of LASV in the environment could be used to further LASV management and mitigation efforts.

Appendix Additional information on environmental persistence and disinfection of Lassa virus.
