# Supplementary material for: Environmental Persistence and Disinfection of Lassa Virus
Source: Emerg Infect Dis. 2023 Nov;29(11):2285–91. doi: 10.3201/eid2911.230678 (PMC10617325; doi:10.3201/eid2911.230678)
Supplement: Appendix — Additional information on environmental persistence and disinfection of Lassa virus. [file 23-0678-Techapp-s1.pdf]

Article DOI: https://doi.org/10.3201/eid2911.230678

EID cannot ensure accessibility for supplementary materials supplied by authors. Readers who have difficulty accessing supplementary content should contact the authors for assistance.

Environmental Persistence and Disinfection of Lassa Virus

Appendix

Wastewater Characterization

Approximately 1 L of primary influent wastewater was aseptically collected from a wastewater treatment plant in northern Indiana, United States, and stored at −80°C before being shipped overnight on ice to Rocky Mountain Laboratories (RML). All physiochemical characterization and chlorine demand testing were conducted at the University of Notre Dame. Appendix Table 1 describes the physiochemical characteristics of the influent wastewater used in the following experiments.

Appendix Table 1. Composition of tested wastewater

Measurements	Test results
pH	6.16
Chemical oxygen demand, mg/L	279
Ammonia, mg/L	29
Nitrate, mg/L	5.0
Phosphorus, mg/L	9.2
Total suspended solids, mg/L	134

Appendix Table 2. Recorded $TCID_{50}$ values for 2 Lassa virus strains on high-density polyethylene over time*

Time, d	Josiah strain	Sauerwald strain
0	868,340.4 (443,997.8)	652,379.4 (462,663.2)
1	22,270.8 (12,317.4)	4,479.2 (1,598.1)
2	694.4 (1,130.9)	592.4 (553.4)
3	6.3 (0)†	632.5 (0)
4	6.3 (0)†	6.3 (0)†
5	6.3 (0)†	6.3 (0)†

*Values are no. (SD). $TCID_{50}$, 50% tissue culture infectious dose.
†Values are at the limit of detection for the $TCID_{50}$ assay.

Appendix Table 3. Recorded $TCID_{50}$ values for 2 Lassa virus strains on stainless steel over time*

Time, d	Josiah strain	Sauerwald strain
0	868,340.4 (443,997.8)	355,655.9 (0)
1	22,270.8 (12,317.4)	13,188.5 (19,379.1)
2	694.4 (1,130.9)	1,124.7 (0)
3	6.3 (0)†	229.3 (349.2)
4	6.3 (0)†	6.3 (0)†
5	6.3 (0)†	6.3 (0)†

*Values are no. (SD). $TCID_{50}$, 50% tissue culture infectious dose.
†Values are at the limit of detection for the $TCID_{50}$ assay.

Appendix Table 4. Recorded $TCID_{50}$ values for 2 Lassa virus strains in deionized water over time*

Time, d	Josiah strain	Sauerwald strain
0	1,834,299.5 (1,300,873.0)	457,487.3 (487,482.5)
1	708,227.6 (252,682.4)	223,961.2 (79,905.2)
2	1,492,835.1 (1,485,653)	316,227.8 (0)
3	398,265.6 (142,093.7)	111,354.0 (61,586.9)
4	316,227.8 (0)	62,619.0 (34,632.9)
5	626,189.7 (346,328.7)	70,822.8 (25,268.2)

*Values are no. (SD). $TCID_{50}$, 50% tissue culture infectious dose/mL.

Appendix Table 5. Recorded $TCID_{50}$ values for 2 Lassa virus strains in wastewater over time*

Time, d	Josiah strain	Sauerwald strain
0	1,518,852.9 (449,339.8)	244,151.8 (124,839.2)
1	239,525.2 (280,422.1)	49,801.9 (440,420.2)
2	39,826.6 (14,209.4)	12,483.9 (12,483.9)
3	95,942.7 (83,987.5)	1,980.2 (1,095.2)
4	8,856.2 (7,828.0)	544.2 (394.8)
5	1,706.1 (1,493.5)	11.14 (6.2)

*Values are no. (SD). $TCID_{50}$, 50% tissue culture infectious dose/mL.

Appendix Table 6. Recorded $TCID_{50}$ values for 2 Lassa virus strains in 0 mg/L sodium hypochlorite over time*

Time, min	Josiah strain	Sauerwald strain
0	1,252,379.4 (692,657.4)	125,237.9 (69,265.7)
0.3	1,678,965.8 (1,670,888.2)	125,237.9 (69,265.7)
1	77,087.0 (61,282.1)	141,645.5 (50,536.5)
5	704,264.7 (389,509.9)	116,011.3 (82,274.4)
15	796,531.2 (284,187.5)	79,653.1 (28,418.7)
30	447,922.4 (159,810.4)	20,000.0 (0)

*Values are no. (SD). $TCID_{50}$, 50% tissue culture infectious dose/mL.

Appendix Table 7. Recorded $TCID_{50}$ values for 2 Lassa virus strains in 1 mg/L sodium hypochlorite over time*

Time, min	Josiah strain	Sauerwald strain
0	1,252,379.4 (692,657.4)	96,060.7 (28,418.7)
0.3	116,011.3 (82,274.4)	3,504.5 (2,846.4)
1	48,830.4 (24,967.8)	2,063.0 (1,463.1)
5	3,037.7 (898.7)	960.6 (284.2)
15	560.1 (495.1)	366.9 (260.2)
30	6.3 (0)†	6.3 (0)†

*Values are no. (SD). $TCID_{50}$, 50% tissue culture infectious dose/mL.
†Values are at the limit of detection for the $TCID_{50}$ assay.

Appendix Table 8. Recorded $TCID_{50}$ values for 2 Lassa virus strains in 5 mg/L sodium hypochlorite over time*

Time, min	Josiah strain	Sauerwald strain
0	1,252,379.4 (692,657.4)	540,188.9 (159,810.4)
0.3	1,124.7 (0)	632.5 (0)
1	632.5 (0)	170.8 (50.5)
5	6.3† (0)	41.7 (61.3), 112.5 (0)‡
15	41.7 (63.1), 112.5 (0)‡	6.3 (0)†
30	6.3 (0)†	6.3 (0)†

*Values are no. (SD). $TCID_{50}$, 50% tissue culture infectious dose/mL.
†Values are at the limit of detection for the $TCID_{50}$ assay.
‡Indicates $\geq$1 values were at the limit of detection; values in parentheses show SD when those values were excluded.

Appendix Table 9. Recorded $TCID_{50}$ values for 2 Lassa virus strains in 10 mg/L sodium hypochlorite over time*

Time, min	Josiah strain	Sauerwald strain
0	2,063,004.8 (1,463,069.4)	222,708.0 (123,173.8)
0.3	632.5 (0)	632.5 (0)
1	540.2 (159.8)	6.3 (0)†
5	6.3 (0)†	6.3 (0)†
15	77.1 (61.3), 112.5 (0)‡	6.3 (0)†
30	6.3 (0)†	6.3 (0)†

*Values are no. (SD). $TCID_{50}$, 50% tissue culture infectious dose/mL.
†Values are at the limit of detection for the $TCID_{50}$ assay.
‡Indicates $\geq$1 values were at the limit of detection; values in parentheses show SD when those values were excluded.

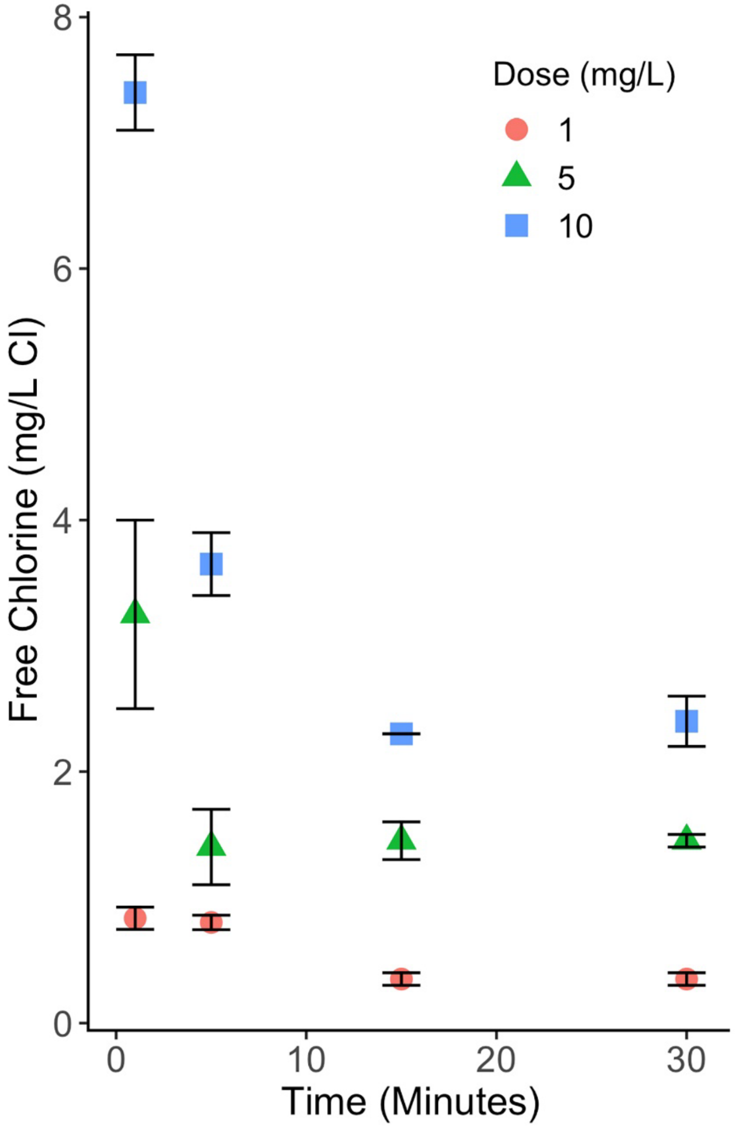

Appendix Figure. Free Chlorine Concentrations for 1, 5, and 10 mg/L of free chlorine as sodium hypochlorite. The y-axis shows the free chlorine concentration as mg/L of Cl determined by the HACH D900 Colorimeter. The x-axis shows the time in minutes. Each data point represents the mean of 3 replicates, and the error bars show the standard deviation.
